# Characterization of the trigeminovascular actions of several adenosine A_2A_ receptor antagonists in an in vivo rat model of migraine

**DOI:** 10.1186/s10194-018-0867-x

**Published:** 2018-05-25

**Authors:** Kristian A. Haanes, Alejandro Labastida-Ramírez, Kayi Y. Chan, René de Vries, Brian Shook, Paul Jackson, Jimmy Zhang, Christopher M. Flores, Alexander H. J. Danser, Carlos M. Villalón, Antoinette MaassenVanDenBrink

**Affiliations:** 1 Division of Vascular Medicine and Pharmacology, Department of Internal Medicine, Erasmus MC, Rotterdam, Dr Molewaterplein 50, 3015 GE Rotterdam, The Netherlands; 2 Janssen Research & Development, L.L.C, Welsh and McKean Roads, Spring House, PA 19477 USA; 3 Departamento de Farmacobiología, Cinvestav-Coapa, Czda. de los Tenorios 235, Col. Granjas-Coapa, Deleg. Tlalpan, C.P, 14330 Ciudad de México, Mexico

**Keywords:** Adenosine receptor, CGS21680, Dural vasodilation, Rat, vasodepressor response

## Abstract

**Background:**

Migraine is considered a neurovascular disorder, but its pathophysiological mechanisms are not yet fully understood. Adenosine has been shown to increase in plasma during migraine attacks and to induce vasodilation in several blood vessels; however, it remains unknown whether adenosine can interact with the trigeminovascular system. Moreover, caffeine, a non-selective adenosine receptor antagonist, is included in many over the counter anti-headache/migraine treatments.

**Methods:**

This study used the rat closed cranial window method to investigate in vivo the effects of the adenosine A_2A_ receptor antagonists with varying selectivity over A_1_ receptors; JNJ-39928122, JNJ-40529749, JNJ-41942914, JNJ-40064440 or JNJ-41501798 (0.3–10 mg/kg) on the vasodilation of the middle meningeal artery produced by either CGS21680 (an adenosine A_2A_ receptor agonist) or endogenous CGRP (released by periarterial electrical stimulation).

**Results:**

Regarding the dural meningeal vasodilation produced neurogenically or pharmacologically, all JNJ antagonists: (i) did not affect neurogenic vasodilation but (ii) blocked the vasodilation produced by CGS21680, with a blocking potency directly related to their additional affinity for the adenosine A_1_ receptor.

**Conclusions:**

These results suggest that vascular adenosine A_2A_ (and, to a certain extent, also A_1_) receptors mediate the CGS21680-induced meningeal vasodilation. These receptors do not appear to modulate prejunctionally the sensory release of CGRP. Prevention of meningeal arterial dilation might be predictive for anti-migraine drugs, and since none of these JNJ antagonists modified per se blood pressure, selective A_2A_ receptor antagonism may offer a novel approach to antimigraine therapy which remains to be investigated in clinical trials.

## Background

Migraine is a neurovascular disorder associated with activation of the trigeminovascular system and release of calcitonin gene-related peptide (CGRP) from trigeminal sensory perivascular nerves, which results in cranial vasodilation and stimulation of sensory nerve transmission [1]. In line with these neurovascular mechanisms: (i) plasma levels of CGRP, which increase during migraine, are normalized by triptans in parallel with amelioration of headache [2]; and (ii) CGRP receptor antagonists [1] and antibodies against CGRP or its receptor [3] are effective in migraine treatment. Although there seem to be some full-responders, the average reduction in migraine days compared to placebo is only in the excess of 1 day per month when administering any CGRP antibody [4]. This limited efficacy resulting from inhibiting CGRP effects suggests that the pathogenesis of migraine could involve additional mechanisms.

Interestingly, adenosine (released centrally and peripherally as a breakdown product of ATP) is another neuromodulator that seems to play a role in migraine pathophysiology [5]. Indeed: (i) adenosine plasma levels have been reported to be increased during migraine attacks [6]; (ii) exogenous adenosine may trigger migraine attacks [7]; (iii) dipyridamole, an adenosine uptake inhibitor, may increase the frequency of migraine attacks [8]; and (iv) an adenosine gene haplotype has been associated with migraine with aura [9]. Accordingly, adenosine receptor antagonists may have potential therapeutic usefulness in the treatment of migraine; while caffeine, a non-selective adenosine receptor antagonist [5], is already present in several over-the-counter anti-headache/migraine medications [10].

The conjunction of structural, transductional and operational criteria has shown that adenosine can activate four subtypes of G-protein-coupled receptors [11, 12], namely adenosine: (i) A_1_ and A_3_ receptors (coupled to G_i_ proteins), which mediate vascular smooth muscle constriction; and (ii) A_2A_ and A_2B_ receptors (coupled to G_s_ proteins), which mediate direct and endothelium-dependent vasodilation [13, 14]. Moreover, the A_1_ receptor can also mediate endothelium-dependent vasodilation [15, 16].

Within this framework, it has been shown ex vivo that adenosine and CGS21680, a stable A_2A_ receptor agonist (with about 10–100-fold selectivity for A_2A_ receptors over A_1_ and A_3_ receptors and poor affinity for A_2B_ receptors [17]), dilate middle meningeal and cerebral arteries respectively, a response blocked by A_2A_ receptor antagonists [13, 18].

The above findings, coupled to the demonstration that the trigeminal ganglion expresses A_2A_ receptors [19] and the ability of this receptor to facilitate CGRP release in the hippocampus [20], beg the questions of whether adenosine A_2A_ receptors can induce meningeal vasodilation in vivo, and also whether they could be involved in neurogenic vasodilation either per se or as modulators of CGRP release in the trigeminovascular system.

**Fig. 1:**
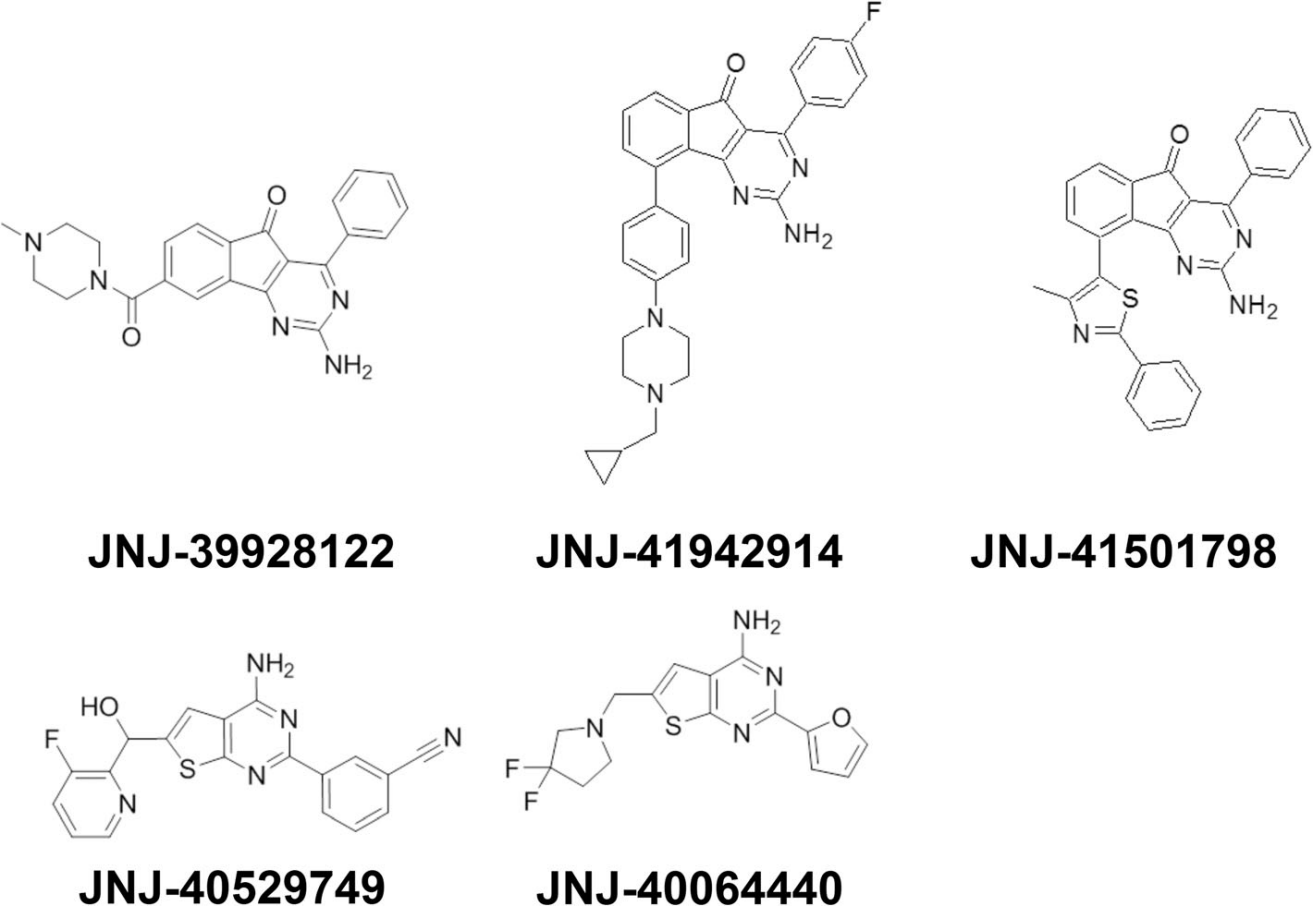
Molecular structures of the JNJ antagonists from Janssen Research & Development

Hence, this study used the rat closed cranial window method, a model predictive of antimigraine action [21], to investigate the effects of five novel adenosine A_2A_ receptor antagonists (Fig. 1) on the vasodilation of the middle meningeal artery produced by either CGS21680 or endogenous CGRP (released by periarterial electrical stimulation). These antagonists (JNJ-41942914, JNJ-39928122, JNJ-40529749, JNJ-40064440 and JNJ-41501798) were developed as described by Shook et al. [22] and display a varying degree of selectivity for A_2A_ over A_1_ receptors (Table 1).

**Table 1:** Affinity constants indicated as IC_50_ in nM (and the corresponding pIC_50_) for the compounds used in the present study

Compound	A_2A_	A_1_	Fold selectivity
			Selectivity A_2A_ vs. A_1_
CGS21680^33^	22 nM (7.6)	3100 nM (5.5)	141
Caffeine^28^	8100 nM (5.1)	20,000 nM (4.7)	2.5
JNJ-39928122^a^	7.9 nM ^λ^ (8.1)	55.1 nM ^λ^ (7.3)	7
JNJ-40529749^a^	4.9 nM (8.3)	89.1 nM (7.1)	18
JNJ-41942914^a^	8.3 nM (8.1)	1093 nM (6.0)	132
JNJ-40064440^a^	8.2 nM (8.1)	1240 nM (5.9)	151
JNJ-41501798^a^	11.5 nM (7.9)	7997 nM (5.1)	695

The JNJ antagonists were developed by Johnson & Johnson Pharmaceutical Research & Development, L.L.C

Hutchison et al. 1989 [33]; Fredholm et al. 1999 [28]; ^a^, Paul Jackson (Janssen Research & Development, personal communication); ^λ^, Indicates K_i_ values

## Methods

### Intravital microscopy experiments

#### Animals

Fifty seven normotensive male Sprague-Dawley rats (300–400 g), purchased from Harlan (Horst, The Netherlands), were maintained at a 12/12-h light-dark cycle (with light beginning at 7 a.m.) and housed at a constant temperature (22 ± 2°C) and humidity (50%), with food and water ad libitum. Only male rats were used to avoid crosstalk between CGRP and hormonal fluctuations during the female oestrus cycle [23]. The animals were anaesthetized with an intraperitoneal (i.p.) injection of sodium pentobarbital (60 mg/kg, followed by 18 mg/kg i.v. per hour when necessary). The adequacy of anaesthesia was judged by a negative tail flick test and the absence of ocular reflexes, amongst others. All experimental protocols of this study were approved by our Institutional Ethics Committee [Erasmus MC; permission protocol number EMC 1931 (118–09-04)], in accordance with the NIH guide for the Care and Use of Laboratory Animals in U.S.A. and the ARRIVE guidelines for reporting experiments in animals [24]. All rats were randomly assigned into the different experimental protocols (see experimental protocol section).

#### General methods

After anesthesia, the trachea was cannulated and connected to a pressure ventilator (small animal ventilator SAR-830 series, CWE Inc., Ardmore, PA, U.S.A.). End-tidal pCO_2_ was monitored (Capstar-100 CWE Inc., PA, U.S.A.) and kept between 35 and 48 mmHg. The left femoral vein and artery were cannulated for intravenous (i.v.) administration of drugs and continuous monitoring of blood pressure, respectively. Two or three samples of blood (at the beginning and at the end of the experiment) were withdrawn via the femoral artery to monitor blood gases and other parameters, which were kept between normal values (pH: 7.35–7.48; pCO_2_: 35–48 mmHg; pO_2_: 100–120 mmHg). The body temperature of each rat was monitored via a rectal thermometer and maintained throughout the experiment (36.5 °C–37.5 °C) by a homeothermic blanket system for rodents (Harvard Instruments, Edenbridge, Kent, U.K.). The rats were placed in a stereotaxic frame and the parietal bone overlying a segment of the dural meningeal artery was carefully drilled thin, applying cold saline (4 °C) until the artery was visible. Since skull drilling induces vasodilation, we allowed the animal to recover for 1 h before the experimental protocol. The drilled area was covered with mineral oil to prevent drying and to facilitate visualization of the meningeal artery. The artery was captured with an intravital microscope (model MZ 16; Leica microsystem Ltd., Heerbrugg, Switzerland) using a cyan blue filter on a cold source of light. A zoom lens (80–450 × magnification) and a camera was used to display images with the blood vessel diameter (30–40 μm at baseline) being continuously monitored and measured with a video dimension analyser (Living Systems Instrumentation Inc., Burlington, VT, U.S.A.). In rats where periarterial electrical stimulation was used to evoke dural vasodilation, a bipolar stimulating electrode (NE 200X, Clark Electromedical, Edenbridge, Kent, U.K.) was placed on the surface of the cranial window approximately within 200 μm from the vessel of interest. The cranial window surface was stimulated at 5 Hz, 1 ms for 10 s (Stimulator model S88, Grass Instruments, West Warwick, RI, U.S.A.). For neurogenic dural vasodilation, we initially started with a current intensity (monitored on an oscilloscope, model 54601A, Hewlett Packard, Palo Alto, CA, U.S.A.) of 100 μA and increased with 50 μA steps until a maximal level of dilatation was achieved, usually at 200 μA. The resulting data were displayed and recorded using a WINDAQ data acquisition system (Version 2.54; DataQ Instruments Inc., Akron, OH, U.S.A.).

#### Experimental protocols

First, 6 animals were used to determine the effect of i.v. adenosine and caffeine on the middle meningeal artery diameter. The doses of adenosine (1 mg/kg) and caffeine (40 mg/kg) were based on previously published work [15, 25]. Further, 51 animals were divided into two groups which received, respectively, periarterial electrical stimulation (150–250 μA; *n* = 27) and the adenosine A_2_ receptor agonist CGS21680 (10 μg/kg, i.v., *n* = 24; the optimal dose as determined in 7 pilot experiments, data not shown). Dural vasodilator responses remained unchanged after repeated treatment for 4 times (data not shown) and in the presence of the vehicle captisol, which was used for dissolving most of the antagonists. Thirty min were allowed between each of these treatments for recovery to the baseline diameter. Subsequently, each of these groups was subdivided into five subgroups (*n* = 3–6 each) which were given (after 30 min) i.v. bolus injections of, respectively, the adenosine A_2A_ receptor antagonists JNJ-41942914 (0.3, 1, and 3 mg/kg), JNJ-39928122, JNJ-40529749, JNJ-40064440 and JNJ-41501798 (all 1, 3 and up to 10 mg/kg). Based on their binding affinities (see Table 1), only doses up until significant blockade, were tested for the CGS21580 response. Each antagonist dose was administered 5 min before periarterial electrical stimulation or CGS21680, except for caffeine (15 min) as previously reported [25]. The duration of each experiment was approximately 2.5 h after stabilization.

#### Data presentation and statistical evaluation

All data are presented as mean ± SEM. The peak increases in dural meningeal artery diameter are expressed as percent change from baseline. Changes in mean arterial blood pressure (MAP) were expressed as absolute values (mm Hg). The difference between the variables within one group was compared by using a one-way repeated measures analysis of variance followed by Dunnet’s test. Dunnet’s test does not give individual *P*-values, hence statistical significance was accepted at *P* < 0.05. When there was only one dose applied (for caffeine), two-tailed paired Student’s T-test was used.

#### Drugs

The compounds used in this study were: sodium pentobarbital (Nembutal; Ceva Sante Animale B.V., Maassluis, The Netherlands); caffeine, adenosine and CGS21680 hydrochloride hydrate (2-p-(2-Carboxyethyl)phenethylamino-5′-N-ethylcarboxamido adenosine hydrochloride hydrate) (Sigma Chemicals Co., Steinheim, Germany); JNJ-41942914, JNJ-39928122, JNJ-40064440, JNJ-40529749 and JNJ-41501798 (gift courtesy from Janssen Research & Development, L.L.C., Raritan, NJ, U.S.A.). Caffeine, adenosine, CGS21680 and JNJ-40064440 were dissolved in distilled water, whereas JNJ-39928122, JNJ-41942914, JNJ-40529749 and JNJ-41501798 were dissolved in captisol (sulfobutylether β-cyclodextrin; Ligand Pharmaceuticals, San Diego, U.S.A.). The suspensions of JNJ-40529749 and JNJ-41501798 were sonicated and filtrated. All solutions were further diluted in saline.

## Results

### General considerations

In order to facilitate the interpretation of the following results, the five JNJ antagonists (Table 1) were sub-divided, a priori, into 3 groups (indicated in different grey-tones): (i) JNJ-39928122 and JNJ-40529749 have ~ 10 fold selectivity for A_2A_ over A_1_ receptors; (ii) JNJ-41942914 and JNJ-40064440 are ~ 100 fold selective for A_2A_ over A_1_ receptors; and (iii) JNJ-41501798 is ~ 700 fold selective for A_2A_ over A_1_ receptors. It is also worth mentioning that caffeine has ~ 2.5 fold selectivity for the rat and ~ 5 fold selectivity for the human A_2A_ vs. A_1_ receptors [K_D_ values, [26]]; however, caffeine also inhibits A_2B_ receptor with similar affinity as for A_1_, which is not the case for the JNJ antagonists.

### Effects of i.v. adenosine and caffeine on dural diameter and MAP

**Fig. 2:**
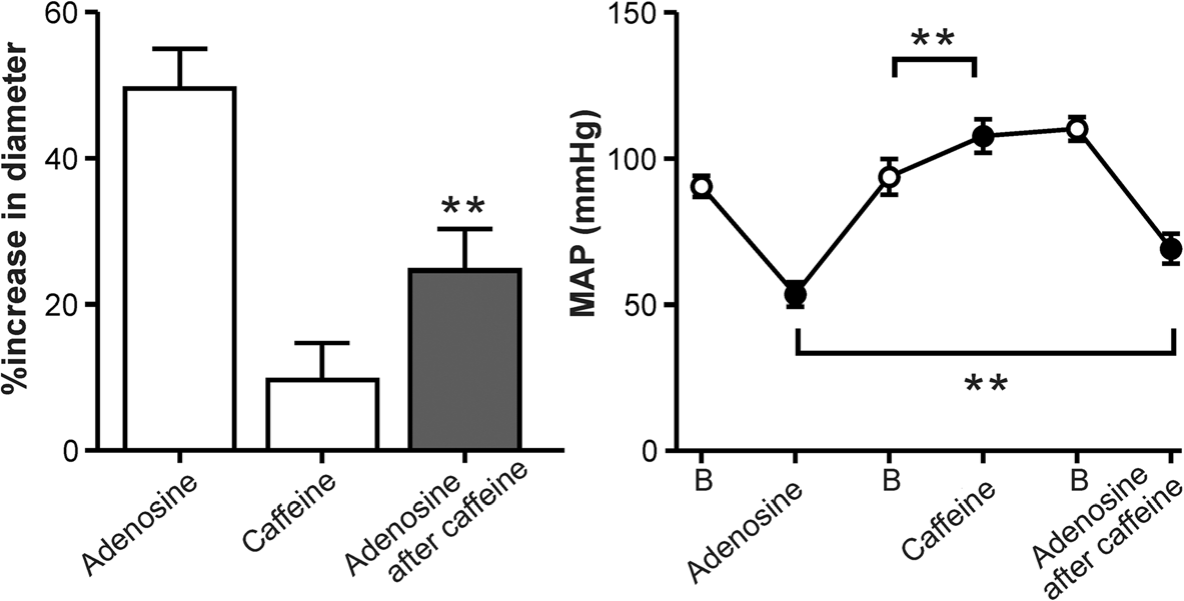
The effect of caffeine on adenosine-induced dural vasodilation. Adenosine (1 mg/kg) was injected i.v. after a recovery period of 30 min. Then, caffeine (40 mg/kg) was injected slowly, and a second adenosine injection (1 mg/kg) was injected 15 min after the caffeine injection (adenosine after caffeine). Left panel illustrates increase in diameter and right panel changes in mean arterial blood pressure, in response to adenosine. Data are ± SEM, *n* = 6, ** *p* < 0.01 compared to the control. Open circles represent baseline measurements before injections, B=Baseline

We initially set out to determine the effect of adenosine on the dural diameter in vivo. Figure 2 shows that (i) 1 mg/kg adenosine caused a dural artery dilation of 50 ± 6% and a drop in blood pressure to 53 ± 4 mmHg; (ii) 40 mg/kg caffeine caused a non-significant dural artery dilation of 12 ± 5%, while blood pressure was increased significantly by 14 ± 3 mmHg; (iii) after a stabilizing period post-caffeine, the second dural artery dilation produced by adenosine was reduced to 25 ± 6% (*n* = 6, *p* = 0.003, which was accompanied by a significantly attenuated drop in blood pressure, to 69 ± 5 mmHg (*p* = 0.004).

### Effect of the JNJ antagonists on the dural dilatation by periarterial electrical stimulation

**Fig. 3:**
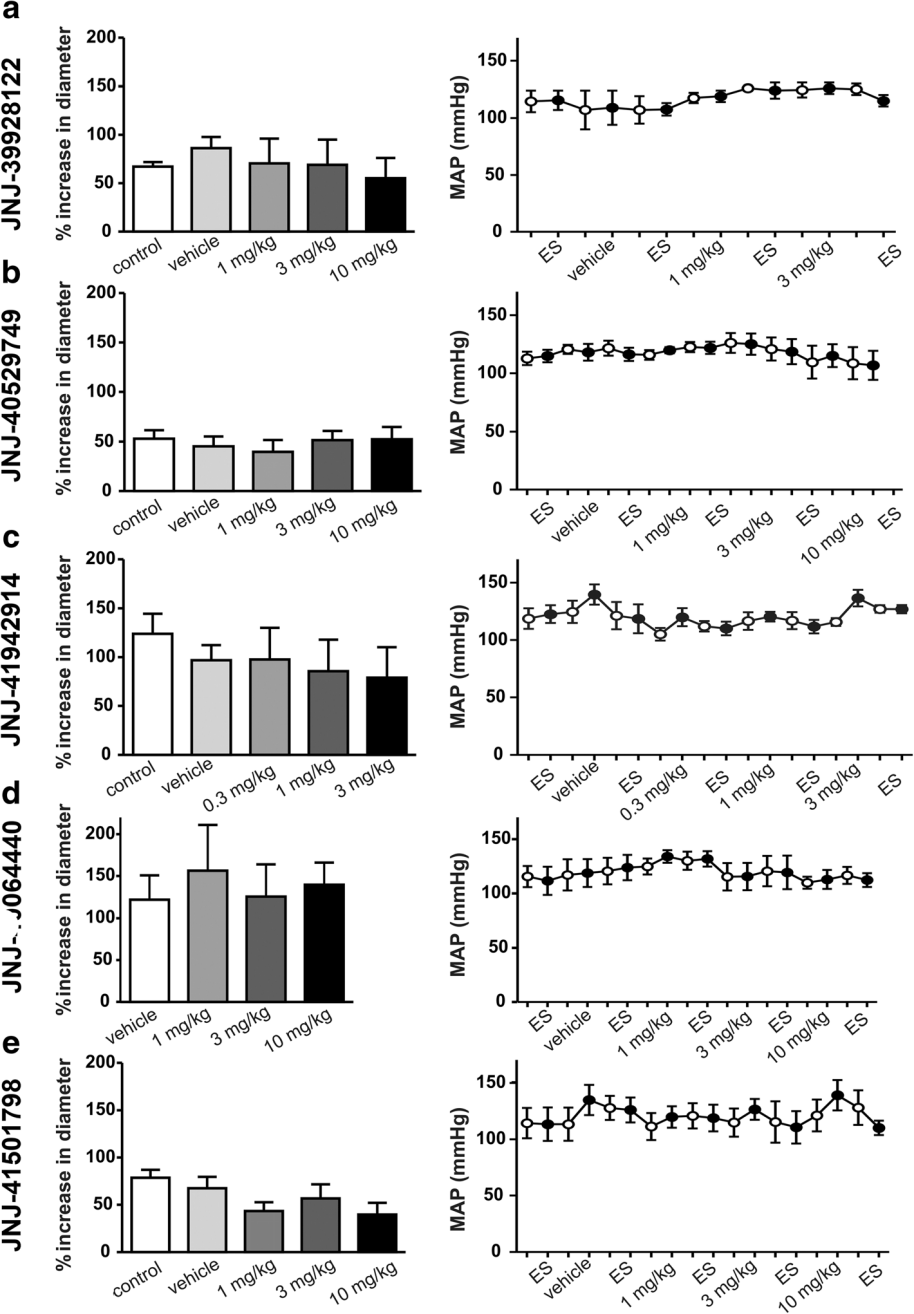
Effect of A_2A_ antagonists on perivascular electrical stimulation of the dural artery. Perivascular electrical stimulation (150–250 μA) in the absence or presence of vehicle, or varying doses of JNJ-39928122 (A, *n* = 4), JNJ-40529749 (B, n = 4–5), JNJ-41942914 (C, n = 6), JNJ-40064440 (D, n = 4), or JNJ-41501798 (E, *n* = 7–8). Data are presented as percentage of increase in diameter, left panels) and changes in mean arterial blood pressure (mm Hg, right panels) induced by periarterial electrical stimulation (ES). Note that none of the treatments produced any significant changes (*p* > 0.05 compared to the vehicle). Open circles represent baseline measurements before injections/ES. JNJ-40064440 was dissolved in water, so vehicle measurements equal control

In order to investigate whether the dural dilation induced by periarterial electrical stimulation could be in part dependent on adenosine release, either as direct activation of vascular adenosine receptors or prejunctional modulation of trigeminal CGRP release, the JNJ antagonists (given i.v.) were investigated in their capability to modify the dural vasodilation produced by electrical stimulation. As shown in Fig. 3 (left panels), neurogenic stimulation induced, overall, an immediate increase in dural artery diameter of 83 ± 7% (*n* = 27). Surprisingly, none of the JNJ antagonists affected this neurogenic vasodilation (left panels). Suggesting that neither A_1_ nor A_2A_ receptors are involved.

### The effect of the JNJ antagonists on MAP before and during neurogenic dural stimulation

As shown in Fig. 3 (right panels), both periarterial electrical stimulation and the JNJ antagonists were devoid of any effect per se on MAP.

### Effects of CGS21680 on dural artery diameter and MAP

Although adenosine A_2A_ or A_1_ receptors did not appear to be important in the vasodilation observed after neurogenic dural stimulation, adenosine vasodilates dural arteries in vivo (Fig. 2), most likely via both A_2A_ and A_2B_ receptors as previously reported ex vivo [13]. Since our study set out to study specifically the role of the adenosine A_2A_ receptor, we continued our study using CGS21680, which is a more biologically stable, highly selective for A_2A_ over A_2B_ receptor agonist [17].

**Fig. 4:**
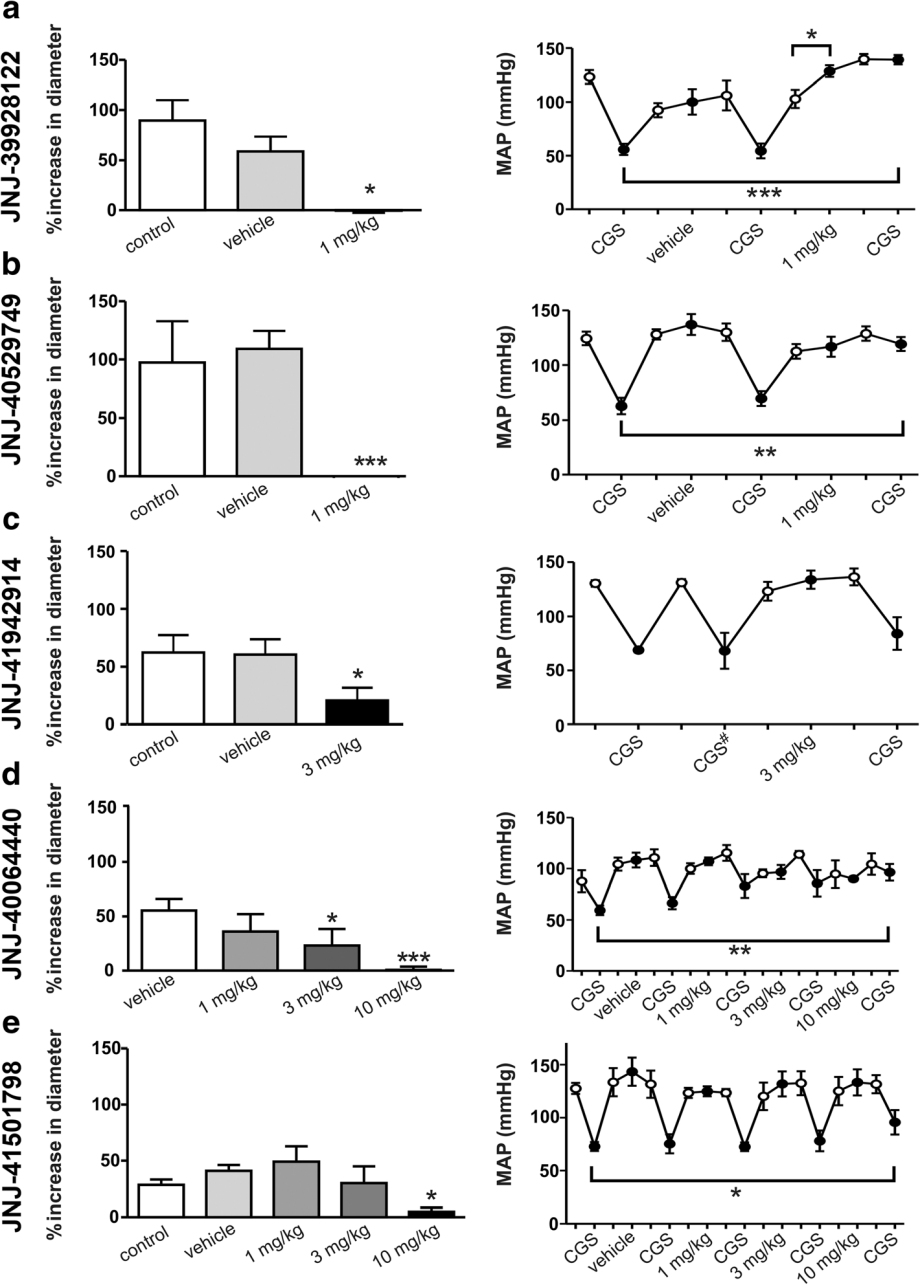
Effect of i.v. CGS21680 on the dural diameter**.** CGS21680 (10 μg/kg) was injected followed by an injection of vehicle and varying doses of JNJ-39928122 (**a)**, *n* = 5, Dunnet critical value: 1014), JNJ-40529749 (**b**), *n* = 3–5, Dunnet critical value: 3791) JNJ-41942914 (**c**), *n* = 4, Dunnet critical value: 6008), JNJ-40064440 (**d**), *n* = 3–4, Dunnet critical value: 8446), or JNJ-41501798 (**e)**, *n* = 5–6, Dunnet critical value: 5848). Data are presented as percentage of increase in diameter (left panels) and changes in mean arterial blood pressure (mm Hg, right panels) induced by CGS21680 (left lower panels). CGS, 10 μg/kg CGS21680 i.v.; * *p* < 0.05, ** *p* < 0.01, *** *p* < 0.001 compared to the vehicle. ^#^CGS in presence of vehicle. Open circles represent baseline measurements before injections. JNJ-40064440 was dissolved in water, so vehicle measurements equal control

As shown in Fig. 4, CGS21680 (10 μg/kg before administration of JNJ antagonists; *n* = 24) mimicked adenosine in its capability to produce: (i) a marked dilation of the dural artery diameter (66 ± 9%; left panels); and (ii) a drop in blood pressure (53 ± 9 mmHg; right panels) and hence excluding the involvement of A_2B_ receptors.

### The lower the selectivity (A_2A_ over A_1_ receptors) the higher the potency of JNJ antagonists to block CGS21680-induced dural vasodilation

To further uncover the nature of the adenosine receptors in the dural vasculature, we explored the effect of the JNJ antagonist with varying selectivity (A_2A_ over A_1_ receptors). Figure 4 (left panels) also shows that all JNJ antagonists significantly blocked the CGS21680-induced dural vasodilation with varying degrees of potency. Specifically, the vasodilation to CGS21680 was: (i) abolished by 1 mg/kg (− 1 ± 2%) of JNJ-39928122 (Fig. 4a); (ii) abolished at 1 mg/kg (− 2 ± 1%) of JNJ-40529749 (Fig. 4b); (iii) significantly attenuated (but not abolished) by 3 mg/kg (21 ± 11%) of JNJ-41942914 (Fig. 4c); (iv) significantly attenuated by 3 mg/kg (23 ± 15%) and abolished (1 ± 3%) by 10 mg/kg of JNJ-40064440 (Fig. 4d); and (v) dose-dependently blocked, and practically abolished by 10 mg/kg (5 ± 4%) of JNJ-41501798 (Fig. 4e). Clearly, the lower the selectivity of A_2A_ vs. A_1_ (Table 1) the higher the potency of JNJ antagonists to block CGS21680-induced dural vasodilation.

### Effect of JNJ antagonists on CGS21680-induced vasodepressor responses

Similarly, the vasodepressor responses to CGS21680 were blocked by the JNJ antagonists as follows: (i) very potently by the less selective antagonists JNJ-39928122 and JNJ-40529749; and (ii) less potently by the highest doses of the more selective antagonists JNJ-41942914, JNJ-40064440 and JNJ-41501798, which display from low to very low affinity for the A_1_ receptor (Table 1).

## Discussion

### Comparison between in vivo and in vitro vascular responses to adenosine

The adenosine receptor antagonists SCH58261 (478-fold A_2A_ over A_1_ selective [27]) and caffeine (non-selective A_1/2A/2B_ [28]) have been shown to block the ex vivo adenosine-induced dilation of endothelium-denuded middle meningeal arteries [18]. In these experiments, not only did caffeine (50 μM) or SCH58261 (1 μM) prevent the dural dilation, but a vasoconstriction to adenosine was unmasked. Interestingly, this effect was not observed in vivo*,* which could be due to the fact that the artery used for the myograph (outer diameter ~ 100 μm) had a larger diameter than in this study (outer diameter ~ 35 μm) and that there potentially are less A_3_ receptors expressed in smaller vessels, as we see no indirect involvement of A_3_ (i.e. vasoconstriction) in the current experiments. These differences require further investigation, but it is known that receptor expression changes along different vascular beds [29].

### General considerations

In addition to the implications discussed below, the present study shows that: (i) both adenosine and CGS21680 produced rat dural vasodilation in vivo; and (ii) for JNJ antagonists, the lower the selectivity (A_2A_ over A_1_ receptors) the higher the potency to block the dural vasodilation and vasodepressor responses induced by CGS21680 (implying that blockade of A_1_ receptors is also necessary to completely block the dural vasodilation in vivo). The latter finding is most likely due to endothelial A_1_ receptors, as the main difference between the in vivo (present study) and the ex vivo studies [18] is the absence of endothelium. Indeed, Honey et al. [21] have shown the presence of adenosine A_1_ receptors mediating vasodilation in the rat middle meningeal artery in vivo.

### The potential role of A_2A_ and A_1_ receptors in the dural vasodilation as prejunctional modulators of neurogenic dural vasodilation or produced by CGS21680

The simplest interpretation of the fact that the JNJ antagonists had no effect on neurogenic dural vasodilation (Fig. 3), which involves CGRP release [1], implies that: (i) adenosine is not released by periarterial electrical stimulation; (ii) A_2A_ receptors do not constitute a positive feedback mechanism for CGRP release, as expected from its transductional properties (positive coupling to G_s_ proteins; [11]); or (iii) cAMP increase, induced by CGRP, is so high that this could have masked the small increase in cAMP levels mediated by A_2A_ receptors [26]. Interestingly, adenosine A_1_ receptors [coupled to G_i_ proteins; [11]] can produce a prejunctional inhibition of the neurogenic dural vasodilation in rats [21]. However, the weakly selective JNJ antagonists (JNJ-39928122 and JNJ-40529749), which would be theoretically expected to block (at least in part) this mechanism, did not increase neurogenic dural vasodilation (Fig. 3).

Several lines of evidence have previously shown in other systems that: (i) the vasodilation produced by adenosine and related agonists is mainly mediated by vascular and endothelial A_2A_ receptors [13, 14] as well as by endothelial A_1_ receptors [16]; and (ii) the trigeminovascular system expresses A_2A_ receptors [19]. In keeping with these findings, our results further demonstrate that the JNJ antagonists blocked CGS21680-induced dural vasodilation (Fig. 4), with a different profile of blockade (dependent on A_2A_ vs. A_1_ selectivity; see below). This reinforces the involvement of adenosine A_2A_ and, probably to a lesser extent, of A_1_ receptors. In addition, based on the poor affinity of CGS21680 for the A_2B_ receptors [17] and similar responses to adenosine, our data did not show any strong involvement of the A_2B_ receptors.

### Systemic effects of JNJ antagonists on A_2A_ and A_1_ receptors

Caffeine is a non-selective adenosine A_1_, A_2A_ and A_2B_ receptor antagonist that does not affect A_3_ receptors at the doses used [28]. Accordingly, caffeine produced a slight increase in blood pressure (Fig. 2), as previously reported [25]. Interestingly, the fact that none of the JNJ antagonists increased blood pressure (Fig. 4, right panel), even at doses that blocked the dural vasodilation to CGS21680 (Fig. 4, left panel) suggests that there is no strong “adenosine vascular tone”. In addition, it is worth emphasizing that A_2B_ receptors are involved in the blood pressure effects of adenosine [30], which would explain the minor difference between caffeine and the JNJ antagonists in our study.

It is well established that A_2A_ receptor agonists lower blood pressure [12, 31]. The A_1_ receptor agonists GR79236 and N6-cyclopentyladenosine (CPA), although less studied, also decrease blood pressure with higher potency than CGS21680, and both cause direct production of endothelial NO [15, 16, 31]. Hence, the vasodepressor response to adenosine in A_1_ −/− mice is reduced [32]. In the present study, the less selective (JNJ-39928122 and JNJ-40529749) A_2A_ vs. A_1_ antagonists potently blocked the decrease in blood pressure, whereas the more selective (JNJ-40064440 and JNJ-41501798) A_2A_ antagonists were less potent, and only effective at 10 mg/kg. These high doses of JNJ-4006440 and JNJ-41501798 also induced inihibition of A_1_ receptors. Blockade of the adenosine A_2A_ and A_1_ receptors prevents systemic vasodilation in response to adenosine, and therefore the block in blood pressure.

### In vivo effects of CGS21680

In binding affinity studies, CGS21680 is 141-fold selective for A_2A_ over A_1_ receptors [33]. However, our study raises the concern whether CGS21680 is a specific A_2A_ receptor agonist in vivo in rats, as it appears that higher blocking affinities for the A_1_ receptor causes a more potent blockade of the vasodepressor and dural vasodilator responses. For the human adenosine receptors, the selectivity for A_2A_ over A_1_ receptors is minimal [34].

The most obvious explanation for the apparent discrepancy between the binding affinity selectively and the in vivo effects, is the location of adenosine receptors, as A_1_ receptors are on the endothelium, whereas the A_2A_ receptors are mainly located on vascular smooth muscle [12]; hence the endothelium will be directly exposed to an apparently higher concentration. In addition, there are opposing findings on the selectivity of CGS21680. For example CGS21680 binds with high affinity (around 1 nM) to A_1_ receptors in the hippocampus of A_2A_−/− mice [35], in contrast, in the same mice CGS21680 had no effect on blood pressure [36].

Comparing our findings with previous studies in rats, the vasodepressor response to CGS21680 (10 μg/kg) was completely blocked by 3 mg/kg of the A_2A_ receptor antagonists ZM241385 [319-fold A_2A_ over A_1_; [15, 27]] or CGS15943 [9-fold A_2A_ over A_1_; [37]]. Clearly, ZM241385 has a higher A_2A_ over A_1_ selectivity, but its K_i_ for A_1_ receptors is 255 nM. Since these binding data are similar to those of our less selective compounds, A_2A_ and also A_1_ receptors would be blocked in these studies.

### Possible clinical implications

On the basis of the above lines of evidence, the antimigraine potential of selective adenosine A_2A_ receptor antagonists would be of particular relevance in those patients whose adenosine plasma levels are markedly increased during a migraine attack. Although our findings indicate that adenosine is not released by perivascular electrical stimulation, inhibition of dural vasodilation is a shared mechanism of current (ergots and triptans) and prospective (CGRP (receptor) antagonists and antibodies) antimigraine drugs [1, 38]. Whether this (antimigraine) mechanism alone is sufficient to attenuate the trigeminal nociceptive transmission associated with migraine headache, remains to be determined. Additionally, other studies have shown that: (i) activation of A_2A_ receptors facilitates the action of CGRP and VIP in the rat hippocampus [20]; (ii) A_2A_ receptor knockout mice are hypoalgesic [36]; and (iii) A_2A_ receptors are expressed in the rat trigeminovascular system [19] as well as in the rat trigeminal ganglion, together with A_1_, A_2B_ and A_3_ receptors [18]. Furthermore, intra-articular administration of adenosine and N6-cyclohexyladenosine (CHA, an adenosine A_1_ receptor agonist), but not CGS21680, significantly increased ketorolac antinociception [39]. These findings, taken together: (i) argue in favor of selective blockade of adenosine A_2_ receptors as a potential antimigraine strategy; and (ii) imply that blockade of A_1_ receptors would be a disadvantage in antimigraine treatment. Obviously, further clinical studies should evaluate the JNJ antagonist(s) with the optimal oral bioavailability based on their pharmacokinetic properties.

## Conclusions

In conclusion, all the JNJ antagonists were capable of blocking CGS21680-induced dural vasodilation without affecting neurogenic dural vasodilation (suggesting no modulation of trigeminal CGRP release). This blockade was more potent when showing lower A_2A_ over A_1_ selectivity, and that both these receptors are involved in the dural artery vasodilation. On this basis, and considering that the JNJ antagonist were devoid of any effect per se on blood pressure, selective A_2A_ receptor antagonism may offer a novel approach to antimigraine therapy that remains to be determined in clinical trials.

## Abbreviations

CGRP: Calcitonin gene-related peptide
i.p.: Intraperitoneal
i.v.: Intravenous
